# A longitudinal study on symptom duration and 60-day clinical course in non-hospitalised COVID-19 cases in Berlin, Germany, March to May, 2020

**DOI:** 10.2807/1560-7917.ES.2021.26.43.2001757

**Published:** 2021-10-28

**Authors:** Neil J. Saad, Felix Moek, Fabienne Steitz, Lukas Murajda, Till Bärnighausen, Thomas Zoller, Kirsten Pörtner, Nadine Muller

**Affiliations:** 1 Department of Infectious Disease Epidemiology, Robert Koch Institute, Berlin, Germany; 2 European Programme for Intervention Epidemiology Training (EPIET), European Centre for Disease Prevention and Control (ECDC), Stockholm, Sweden; 3 Postgraduate Training for Applied Epidemiology, Robert Koch Institute, Berlin, Germany; 4 Local Health Authority Berlin-Mitte, Berlin, Germany; 5 Heidelberg Institute of Global Health, Medical Faculty and University Hospital, Heidelberg University, Heidelberg, Germany; 6 Harvard Center for Population and Development Studies, Harvard University, Cambridge, United States; 7 Department of Global Health and Population, Harvard School of Public Health, Boston, United States; 8 Department of Infectious Diseases and Respiratory Medicine, Charité – Universitätsmedizin Berlin, corporate member of Freie Universität Berlin, Humboldt-Universität zu Berlin, and Berlin Institute of Health, Berlin, Germany

**Keywords:** clinical epidemiology, prevalence, prospective study, signs and symptoms, public health surveillance, disease outbreak, COVID-19, SARS-CoV-2

## Abstract

**Background:**

Detailed information on symptom duration and temporal course of patients with mild COVID-19 was scarce at the beginning of the COVID-19 pandemic.

**Aim:**

We aimed to determine the longitudinal course of clinical symptoms in non-hospitalised COVID-19 patients in Berlin, Germany.

**Methods:**

Between March and May 2020, 102 confirmed COVID-19 cases in home isolation notified in Berlin, Germany, were sampled using total population sampling. Data on 25 symptoms were collected during telephone consultations (a maximum of four consultations) with each patient. We collected information on prevalence and duration of symptoms for each day of the first 2 weeks after symptom onset and for day 30 and 60 after symptom onset.

**Results:**

Median age was 35 years (range 18–74), 57% (58/102) were female, and 37% (38/102) reported having comorbidities. During the first 2 weeks, most common symptoms were malaise (94%, 92/98), headache (71%, 70/98), and rhinitis (69%, 68/98). Malaise was present for a median of 11 days (IQR 7–14 days) with 35% (34/98) of cases still reporting malaise on day 14. Headache and muscle pain mostly occurred during the first week, whereas dysosmia and dysgeusia mostly occurred during the second week. Symptoms persisted in 41% (39/95) and 20% (18/88) of patients on day 30 and 60, respectively.

**Conclusion:**

Our study shows that a significant proportion of non-hospitalised COVID-19 cases endured symptoms for at least 2 months. Further research is needed to assess the frequency of long-term adverse health effects in non-hospitalised COVID-19 patients.

## Background

The coronavirus disease (COVID-19) pandemic caused by the severe acute respiratory syndrome coronavirus 2 (SARS-CoV-2) emerged in Wuhan, China in December 2019 and has spread across the globe. As infections peaked across many countries, more has become known about the epidemiological features of the pandemic, the characteristics of those infected and the risk factors associated with hospital or intensive care unit (ICU) admissions and deaths [1-3].

However, detailed investigations on the temporal course of COVID-19 symptoms during the early stages of the pandemic have mostly focused on hospitalised patients or patients admitted to ICU even though the vast majority of infected patients do not require inpatient treatment. The characteristics and duration of symptoms of these patients, who are often referred to as having a mild course of disease and who mostly stay in home isolation or in community health centres during the acute phase of disease, have more recently become the subject of investigation [4-9]. These recent findings provide evidence that a subset of patients, including those with a mild course of disease, report persistent symptoms that result in long-term adverse health effects [4-9].

The aim of this study was to investigate the prevalence, duration and temporal dynamics of clinical symptoms in non-hospitalised COVID-19 patients to better understand the clinical spectrum and disease duration among this group of COVID-19 patients at the onset of the pandemic.

## Methods

### Study population

The target population for this study was non-hospitalised adults with confirmed SARS-CoV-2 infection who were consecutively notified to the local health authority (LHA) in Mitte, a central district of Berlin, Germany. Additional information on the study area and setting is presented in Supplement S1.

The study period was between 30 March and 9 April 2020 and between 13 April and 20 May 2020; the interruption was due to a public holiday. Inclusion criteria were defined as follows: (i) PCR-confirmed SARS-CoV-2 infection notified to the LHA of Berlin, district Mitte; (ii) case notification to the LHA not later than day 9 after symptom onset (SO) (to reduce recall bias); (iii) patient age at least 18 years; (iv) not hospitalised for COVID-19 infection during the first 14 days after SO; and (v) reachable by telephone (to enable participation). Case verification for eligibility and inclusion of participants occurred prospectively on a daily basis and was based on the official LHA notification registry. In total, 130 cases met the inclusion criteria. Of these, 28 cases had to be excluded for reasons such as not consenting to participate, dementia, or speaking insufficient German or English, leaving 102 cases included in the study. The sampling process is further described in Supplement S2.

During the study period, SARS-CoV-2 testing was recommended for individuals either experiencing new symptoms of any kind who had an epidemiological link to a PCR-confirmed SARS-CoV-2 case or experiencing symptoms considered compatible with COVID-19 at the time. Testing of asymptomatic individuals was generally discouraged.

### Data collection

A structured questionnaire on the prevalence and temporal variation of 25 symptoms was developed by the authors based on the available literature at the onset of the pandemic (Supplement S3). Data collection was performed by repeated telephone consultation by clinically-experienced physicians, who were also epidemiologists. For each participant, all consultations were scheduled individually based on the participant’s symptom onset (SO) date:

- The first call was made between day 7 and 10 after SO to assess retrospectively the prevalence of symptoms for each day from day 1 to the day of the call.
- The second call was made between day 14 and 18 to assess retrospectively the prevalence of symptoms for each day after the first call up to days 14 through 18.
- The third call was made between day 28 and 35 to assess the prevalence of symptoms one month after SO (day 30).
- The fourth call was made between day 58 and 70 to assess the prevalence of symptoms 2 months after SO (day 60).

As we assumed that symptoms did not recur once resolved on day 30, follow-up consultations on day 60 after SO (the fourth call) were only made for participants reporting persistent symptoms on day 30 after SO (during the third call).

Definitions and lay explanations of symptoms were agreed upon by the principal investigators (Supplement S4). The perception of disease severity was assessed by collecting data on severity of malaise (mild, moderate, severe). Malaise was defined as a general feeling of illness. On day 60, we asked the cases whether their physical performance capacity had returned to their pre-SARS-CoV-2 levels and whether they had consulted a medical doctor for persisting symptoms since day 30. Additional data on sex, comorbidities, smoking, weight, height and healthcare worker status were collected.

### Data quality assurance and statistical analysis

A standardised data entry mask including data checks for completeness and limits was configured in EpiData v3.1 [10]. Data quality was assured by duplicate entry and validation by the study team. Anonymity of study participants was ensured by restricting personal identifiable information to the physician responsible for the telephone consultation, and data were anonymised at the data entry level. As continuous variables were not normally distributed, medians and interquartile ranges (IQR) were calculated. To evaluate the clinical presentation of COVID-19, we grouped symptoms according to the following symptom categories: (i) respiratory, (ii) gastrointestinal, (iii) systemic illness, (iv) neurological, and (iv) other (symptoms belonging to none of the four preceding groups). Differences between sexes were tested using a Wilcoxon rank sum test for continuous variables, and a chi-squared test or Fisher’s exact test, as appropriate, for categorical variables. Statistical significance was defined as p < 0.05. All analyses were performed in R using RStudio (R Foundation for Statistical Computing, Vienna, Austria, and RStudio, Boston, United States of America).

### Ethical statement

This study was conducted by the LHA Berlin (district Mitte) within the framework of the German Protection against Infection Act and in response to the COVID-19 pandemic [11]. In Germany, local health authorities are obliged to collect information on clinical symptoms of notifiable diseases for routine surveillance. Thus, institutional review was not sought. No biological materials were collected. Participation was voluntary and verbal informed consent was given before the start of the interviews. Verbal informed consent included informing the participants about the purpose and extent of the study, that participation was voluntary, that their anonymity would be ensured, and that they could terminate their participation or retract collected personal data at any time. Support from the national public health institute – i.e., the Robert Koch Institute (RKI) – to the LHA was provided after official request.

## Results

### Study population

Of the 102 study participants, four cases had an asymptomatic disease course (follow-up stopped 14 days after notification). Of the remaining 98 cases reporting symptoms, 95 (97%) could be reached for repeat follow-up until day 30. Of the 39 (41%) cases reporting persisting symptoms on day 30, 32 (82%) could be reached for follow-up 60 days after SO (Supplement S2).

The study participants were predominately female (57%, 58/102), had a median age of 35 years (IQR: 29–47) and the majority (72%, 73/102) had never smoked. Healthcare staff comprised 47% (47/100) of the cases. A total of 37% (38/102) reported comorbidities, obesity (body mass index ≥ 30) being the most common (15%, 14/95). Other than hypothyroidism being more prevalent in female participants (p = 0.04), we did not observe any other significant differences between the sexes for these factors (Table 1).

**Table 1:** Sociodemographic characteristics and comorbidities among non-hospitalised COVID-19 cases, Berlin, Germany, March–May, 2020 (N = 102)

Variable	Overall^a^ N = 102		N	Female^a^ n = 58		Male^a^ n = 44		p value^b^
Age in years, median (IQR)	35 (29–47)		102	38 (30–51)		34 (30–41)		0.10
	n	%	Total	n	%	n	%	
Pregnant	5	4.9	102	5	8.6	NA	NA	NA
Smoking								
Never smoker	73	72	102	41	71	32	73	> 0.9
Ex-smoker	12	12		7	12	5	11	
Active smoker	17	17		10	17	7	16	
Pack-years, median (IQR)	8	(2–14)	29	10	(5–15)	4	(2–8)	0.08
Healthcare staff^c^	47	47	100	26	46	21	49	> 0.9
Any comorbidity^d^	38	37	102	27	47	11	25	0.04
Obesity^e^	14	15	95	9	16	5	12	0.8
Hypothyroidism	10	9.8	102	9	16	1	2.3	0.04
Arterial hypertension	7	6.9	102	5	8.6	2	4.5	0.7
Asthma	7	6.9	102	6	10	1	2.3	0.14
Psychiatric conditions	6	5.9	102	4	6.9	2	4.5	0.7
Cardiac diseases	5	4.9	102	3	5.2	2	4.5	> 0.9
Hyperlipoproteinaemia	3	2.9	102	3	5.2	0	0	0.3
Diabetes	2	2.0	102	2	3.4	0	0	0.5
Neurological diseases	2	2.0	102	2	3.4	0	0	0.5
Skin conditions	2	2.0	102	1	1.7	1	2.3	> 0.9
COPD	1	1.0	102	1	1.7	0	0	> 0.9
Renal insufficiency	0	0	102	0	0	0	0	NA
Other comorbidities	9	8.8	102	7	12	2	4.5	0.3

COPD: chronic obstructive pulmonary disease; COVID-19: coronavirus disease; IQR: interquartile range; NA: not applicable.

^a^ Number and %, if not indicated otherwise.

^b^ Statistical tests performed: Wilcoxon rank-sum test; Fisher's exact test; chi-squared test of independence.

^c^ Healthcare staff include professionals working in the direct environment of patients (e.g., nurses, midwives, physiotherapists, medical doctors, and cleaning staff). Data not provided by two cases.

^d^ 12 cases had more than one comorbidity.

^e^ Obesity was defined as a body mass index ≥ 30. It could not be calculated for seven patients as data on either weight or height was not provided.

### Prevalence of symptoms and long-term follow-up

Nearly all cases (94%, 92/98) experienced malaise at least once in the 2 weeks after SO. Other common symptoms were headache (71%, 70/98), rhinitis (69%, 68/98), muscle pain (65%, 64/98), cough (64%, 63/98) and dysosmia (60%, 59/98). Dyspnoea and fever, symptoms commonly included in case definitions of COVID-19, were only reported by 29% (28/98) and 39% (38/98) of the study participants, respectively (Table 2).

**Table 2:** Prevalence of symptoms at any time between day 1 and 14 (n = 98), at day 30 (n = 95) and at day 60 (n = 88) after symptom onset in non-hospitalised COVID-19 cases, Berlin, Germany, March–May, 2020

	Day 1–14 n = 98		Day 30 n = 95		Day 60 n = 88^a^	
	n	%	n	%	n	%
Any symptom^b^	98	100	39	41	18	20
Malaise	92	94	7	7.4	3	3.4
Respiratory symptoms						
Rhinitis	68	69	3	3.2	2	2.3
Cough	63	64	11	12	3	3.4
Sore throat	47	48	3	3.2	1	1.1
Dyspnoea	28	29	3	3.2	3	3.4
Pleuritic pain	9	9.2	0	0	0	0
Hoarseness	8	8.2	0	0	0	0
Gastrointestinal symptoms						
Diarrhoea	27	28	2	2.1	0	0
Nausea	27	28	1	1.1	1	1.1
Abdominal pain	17	17	1	1.1	0	0
Vomiting	6	6.1	0	0	0	0
Symptoms of systemic illness						
Muscle pain	64	65	0	0	1	1.1
Fatigue	53	54	5	5.3	3	3.4
Fever	38	39	0	0	0	0
Chills	31	32	0	0	0	0
Neurological symptoms						
Headache	70	71	6	6.3	2	2.3
Dysosmia	59	60	21	22	12	14
Dysgeusia	45	46	8	8.4	5	5.7
Hyperesthesia	17	17	0	0	0	0
Numbness	5	5.1	0	0	0	0
Other symptoms						
Back pain	36	37	3	3.2	1	1.1
Joint pain	20	20	0	0	1	1.1
Chest tightness	16	16	3	3.2	1	1.1
Listlessness	16	16	2	2.1	0	0
Chest pain, unspecific	5	5.1	1	1.1	0	0
Rash	6	6.1	0	0	0	0
Conjunctivitis	3	3.1	0	0	0	0

COVID-19: coronavirus disease.

^a^ We assumed resolved symptoms did not recur in the long term. Therefore, patients with resolved symptoms on day 30 were assumed to be symptom-free on day 60.

^b^ Symptoms were grouped into symptom categories by the physicians conducting the telephone interviews. Cases can have multiple symptoms simultaneously between day 1 and 14, at day 30, or at day 60.

We also found that 51% (50/98) of the cases studied consumed antipyretics during the first 2 weeks for a median of 3 days (IQR: 1–6 days). We observed some differences between sexes: dysgeusia (54% vs 34%, p=0.075) and nausea (39% vs 12%, p = 0.008) were more common among females than males (Supplement S5).

In total, 41% (39/95) still experienced at least one symptom on day 30, with dysosmia and cough being the most common symptoms persisting 1 month after SO. On day 60, 20% (18/88) of the cases were still symptomatic, with dysosmia being the most commonly reported symptom (14%, 12/88) (Table 2). Moreover, when cases experienced symptoms at day 30 or day 60, nearly all had already experienced the symptoms at least once after SO (Supplement S6).

In addition to the symptoms described above, 8.0% (7/88) of all cases reported having reduced physical performance capacity on day 60 compared to their pre-COVID-19 physical performance capacity. Furthermore, 10% (9/88) of cases reported having consulted medical advice because of persisting or worsening symptoms between day 30 and 60. None of these individuals required hospitalisation during that period.

### Temporal variation of malaise and other symptoms

**Figure 1:**
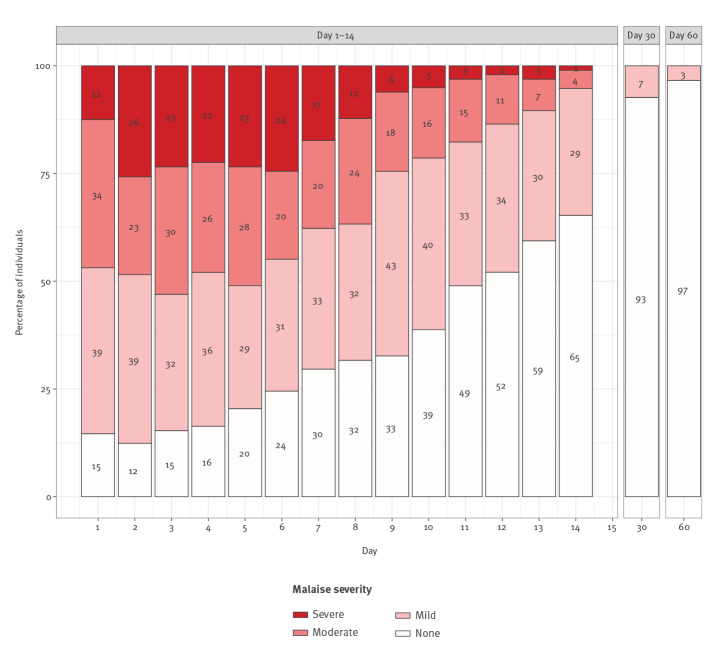
Perception of severity of malaise in the 2 weeks after symptom onset in non-hospitalised COVID-19 cases, Berlin, Germany, March–May, 2020

The cases’ subjective assessment of feeling ill was reflected by the perception and severity of malaise in the 2 weeks after SO. In the first week of illness, approximately half of the cases reported severe or moderate malaise on a daily basis. After the first week, the number of cases having severe and moderate malaise decreased. However, 52% (50/96) needed more than 11 days for their malaise to subside. Two weeks after SO, 35% (33/95) of the patients still reported some, predominately mild, malaise. Feeling ill lingered in a minority of cases; 7.4% (7/95) and 3.4% (3/88) of them still reported feeling ill on day 30 and 60 after SO, respectively (Figure 1).

COVID-19: coronavirus disease.

We assumed resolved malaise did not recur in the long term. Therefore, patients with resolved malaise on day 30 were assumed to be malaise-free on day 60.

Study population size: n = 98 between day 1 and 14, n = 95 at day 30, and n = 88 at day 60.

**Figure 2:**
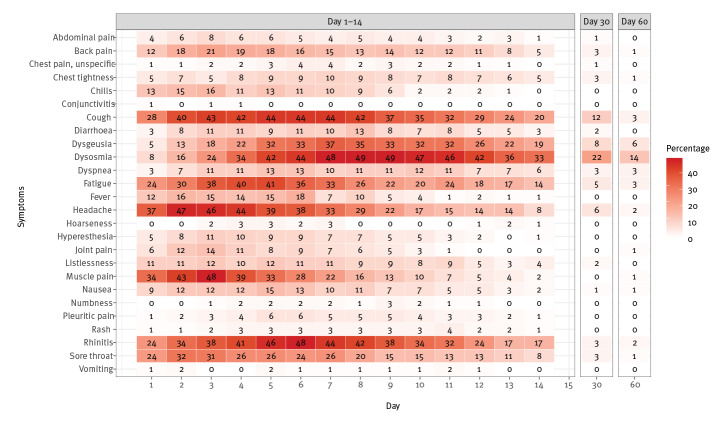
Heatmap depicting the temporal variation of symptoms in the first 2 weeks after symptom onset in non-hospitalised COVID-19 cases, Berlin, Germany, March–May, 2020

As previously noted, the majority of cases did not experience all the symptoms associated with COVID-19. Moreover, the timing of when symptoms were present varied: rhinitis or cough seemed to occur throughout the 2 weeks after SO. However, the peak of cases experienced rhinitis or cough on day 5 or day 6 after SO, whereas the peak of dysosmia and dysgeusia was between day 7 and day 9. Dyspnoea was not commonly prevalent and occurred mostly between day 2 and day 11. Other symptoms such as chest tightness, chest pain or pleuritic pain occurred rarely in the 2 weeks after SO. Fever and headache, on the other hand, seemed to be present mostly in the first week; however, relatively few individuals experienced fever (Figure 2).

COVID-19: coronavirus disease.

Study population size: n = 98 between day 1 and 14, n = 95 at day 30, and n = 88 at day 60.

### Diversity and duration of symptoms in the first 2 weeks of illness

The clinical presentation of COVID-19 among the study population was diverse; more than half (54%, 53/98) of those symptomatic in the 2 weeks after SO reported at least eight different symptoms. We found that 65% (64/98) reported having symptoms from at least four different symptom groups in the 2 weeks after SO.

**Figure 3:**
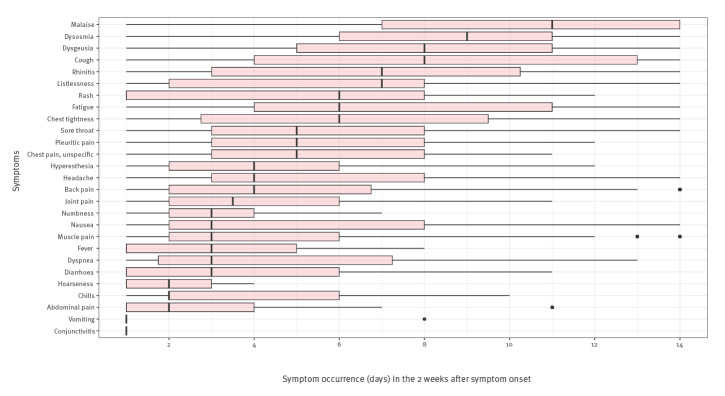
Duration of specific symptoms in the 2 weeks after symptom onset in non-hospitalised COVID-19 cases, Berlin, Germany, March–May, 2020 (n = 98)

During the first 2 weeks of illness, malaise was present the longest (median: 11 days, IQR: 7–14 days). Other symptoms of longer duration were dysosmia (median: 9 days, IQR: 6–11 days), dysgeusia (median: 8 days, IQR: 5–11 days), and cough (median: 8 days, IQR: 4–13 days). Symptoms of intermediate duration were listlessness (median: 7 days, IQR: 2–8 days) and rhinitis (median: 7 days, IQR: 3–10 days). The median duration of all other symptoms was less than 7 days; for example, dyspnoea, fever and muscle pain were only present for a median of 3 days (Figure 3). Most patients could recall their symptoms very well; however, 4–9% of participants could not recall their clinical course of dysosmia on a day-to-day basis.

COVID-19: coronavirus disease.

Duration of symptoms shown using boxplots. Boxes denote 25th to 75th percentile with the 50th percentile marked by a vertical black line inside the box.

COVID-19 symptoms persisted for the entire 2 weeks after SO for the majority of cases; 59% (58/98) experienced at least one symptom every day for 2 weeks. In first 2 weeks after SO, 7% (7/98) had an intermittent clinical course (i.e., a symptom-free period interrupted by a symptomatic period), and 34% (33/98) became and remained symptom-free after an initial symptomatic period. For these cases, only 7 of 33 recovered within one week after SO. The majority of patients (61%, 20/33) needed more than 11 days to become symptom-free.

## Discussion

This study focused on duration and temporal variation of symptoms in non-hospitalised COVID-19 cases in Berlin, Germany. The most common symptoms were malaise, headache, and rhinitis. One month after SO 41% of the cases reported symptoms and 2 months after SO 20% of the cases reported symptoms.

The detailed analysis of the duration of symptoms in the 2 weeks after SO showed that malaise, dysosmia, dysgeusia and cough were present the longest (median duration 8–11 days). However, because the daily symptom assessment during the first two-week period was right-censored at day 14, the symptom duration might be underestimated.

The most reported symptoms during the first 2 weeks of illness were non-specific symptoms such as malaise, headache and muscle pain as well as respiratory symptoms such as rhinitis and cough. The clinical picture found in this study differs from most hospitalised cohorts, where headache, muscle pain and rhinitis were less commonly reported but dyspnoea and fever were more frequently observed [12-14]. We found that both headache and muscle pain were mostly present during the first week after SO. As the median duration between SO and hospitalisation is between 4 and 5 days, these symptoms might already have disappeared by the time the individual’s condition was deteriorating [1,12]. The fact that 50% of cases consumed antipyretics might explain the comparably low prevalence of fever observed in our study population.

As with other investigators [15,16], we observed a wide clinical variety of symptoms: 53% of cases complained of more than eight different symptoms in the 2 weeks after SO. As a consequence, patients often presented with an unspecific clinical picture where gastrointestinal, respiratory and neurological symptoms overlapped. These findings underline the difficulty in formulating a sufficiently sensitive but workable clinical case definition. Furthermore, the diverse clinical presentation strengthens the importance of laboratory confirmation to establish the diagnosis of SARS-CoV-2 infection, both for clinical management as well as for public health action intended to contain further spread of disease [17,18].

From a public health point of view, prolonged symptoms among COVID-19 cases might create uncertainty about when to conclude home isolation [19]. This might lead to an increased workload for public health authorities as well as frustration and possibly negative psychological effects for infected individuals. Our findings emphasise that having mild COVID-19 can have relevant health implications for at least 2 months after disease onset in younger adults, who have a low prevalence of pre-existing conditions. Although longitudinal studies with longer follow-up are under way to investigate the long-term health effects following COVID-19, our findings can serve as early guidance for physicians treating COVID-19 outside a hospital setting.

We describe in detail the duration of specific symptoms including a 30- and 60-day follow-up in non-hospitalised COVID-19 patients in the early phase of the COVID-19 pandemic. Symptoms most commonly reported after 2 months were malaise, dysosmia, dysgeusia, cough and dyspnoea, most of which were also more frequent from the onset. Gastrointestinal symptoms did not appear to persist over a longer period. As this study did not include questions on cognitive functions, possible detrimental COVID-19 effects on cognitive health cannot be excluded and have recently been reported by other studies [20,21]. The findings on symptom persistence 2 months after SO, including reduced physical performance capacity for a substantial subset of patients, are concerning as they add to a growing body of evidence that acute COVID-19 can result in prolonged illness even among individuals with mild disease, which has been confirmed over time [22-25]. Our results underscore the need for studies with extended follow-up to assess the long-term effects of COVID-19.

Current investigations mostly assess the prevalence and duration of symptoms of COVID-19 based on populations sampled in hospitals or care facilities. In comparison, our study population was sampled in direct collaboration with the LHA of the study area and based on the LHA’s official notification registry. As the German Protection against Infection Act requires that both direct care providers and diagnostic laboratories notify the LHA of the residence of every suspected and PCR-confirmed case of COVID-19 within 24 hours [11], the total target population should be represented by the study population. Second, the study was performed in an urban setting where free testing had been offered since the early phase of the pandemic, and several testing sites were actively promoted (e.g., hospitals, outpatient departments, drive-by testing centres, and general practitioners’ offices) [26]. Therefore, we assume a low level of, if any, physical or financial barriers towards healthcare seeking and diagnosis for residents in the district.

Our study participants were generally younger and healthier than cohorts from other studies. As for older age, comorbidities such as hypertension, pre-existing cardiac conditions, chronic obstructive pulmonary disease, and diabetes are confirmed risk factors for a more severe disease course and hospitalisation [12,13].

Our study has some limitations. Despite low physical and financial barriers towards SARS-CoV-2 testing in the study district, we cannot entirely exclude that psychological or cultural barriers might have influenced the acceptance of free diagnostics and therefore the representativeness of the study population. Furthermore, the composition of our study population might be influenced by the recommended testing strategy for SARS-CoV-2 infection. With the exemption of outbreak investigations and without an epidemiological link, testing of asymptomatic patients with atypical COVID-19 symptoms was not recommended in Germany at the time. Therefore, we assume that individuals with very mild or asymptomatic disease are underrepresented. Moreover, because our participants were asked about their symptoms retrospectively by trained physicians, we cannot exclude the possibility that recall and attention bias influenced our findings – e.g., participants might remember their symptoms better because they are being interviewed. However, we believe that our results resemble those of a thorough and systematic history taken by a physician in routine clinical settings.

### Conclusion

Our findings illustrate that non-hospitalised cases with a mild course of COVID-19 experience a multitude of symptoms over a prolonged period. These patients might not be able to fully engage in working activities for a long period and/or might need assistance and care from their relatives, possibly resulting in negative economic effects to the individual as well as to society as a whole. Further research is needed to assess the frequency and prevention of long-term adverse health effects in these patients. Finally, the clinical presentation of non-hospitalised COVID-19 cases from our study partly differs from symptoms used for case-finding activities in the early phase of the pandemic. With the attempt to capture as many cases as possible to guide decision-making, symptoms in case definitions might need to be adapted to better cover COVID-19 outpatients.

## Supplementary Data

762000 Supplementary Material

## References

[1] Cummings MJ Baldwin MR Abrams D Jacobson SD Meyer BJ Balough EM Epidemiology, clinical course, and outcomes of critically ill adults with COVID-19 in New York City: a prospective cohort study. Lancet. 2020;395(10239):1763-70. 10.1016/S0140-6736(20)31189-2 32442528 PMC7237188

[2] Karagiannidis C Mostert C Hentschker C Voshaar T Malzahn J Schillinger G Case characteristics, resource use, and outcomes of 10 021 patients with COVID-19 admitted to 920 German hospitals: an observational study. Lancet Respir Med. 2020;8(9):853-62. 10.1016/S2213-2600(20)30316-7 32735842 PMC7386882

[3] Yang R Gui X Xiong Y. Patients with respiratory symptoms are at greater risk of COVID-19 transmission. Respir Med. 2020;165:105935. 10.1016/j.rmed.2020.105935 32308203 PMC7151456

[4] Augustin M Schommers P Stecher M Dewald F Gieselmann L Gruell H Post-COVID syndrome in non-hospitalised patients with COVID-19: a longitudinal prospective cohort study. Lancet Reg Health Eur. 2021;6:100122. 10.1016/j.lanepe.2021.100122 34027514 PMC8129613

[5] Blomberg B Mohn KG-I Brokstad KA Zhou F Linchausen DW Hansen B-A Long COVID in a prospective cohort of home-isolated patients. Nat Med. 2021;27(9):1607-13. 10.1038/s41591-021-01433-3 34163090 PMC8440190

[6] Goërtz YMJ Van Herck M Delbressine JM Vaes AW Meys R Machado FVC Persistent symptoms 3 months after a SARS-CoV-2 infection: the post-COVID-19 syndrome? ERJ Open Res. 2020;6(4):00542-2020. 10.1183/23120541.00542-2020 33257910 PMC7491255

[7] Johnsen S Sattler SM Miskowiak KW Kunalan K Victor A Pedersen L Descriptive analysis of long COVID sequelae identified in a multidisciplinary clinic serving hospitalised and non-hospitalised patients. ERJ Open Res. 2021;7(3):00205-2021. 10.1183/23120541.00205-2021 34345629 PMC8091683

[8] Petersen MS Kristiansen MF Hanusson KD Danielsen ME Á Steig B Gaini S Long COVID in the Faroe Islands - a longitudinal study among non-hospitalized patients. Clin Infect Dis. 2020;ciaa1792. 10.1093/cid/ciaa1792 33252665 PMC7799340

[9] Stavem K Ghanima W Olsen MK Gilboe HM Einvik G. Persistent symptoms 1.5-6 months after COVID-19 in non-hospitalised subjects: a population-based cohort study. Thorax. 2021;76(4):405-7. 10.1136/thoraxjnl-2020-216377 33273028 PMC7716295

[10] Lauritsen JM, Bruus M. EpiData (version 3.1). A comprehensive tool for validated entry and documentation of data. The EpiData Association. [Accessed: 1 Mar 2020]. Available from: https://epidata.dk

[11] Bundesministerium der Justiz und für Verbraucherschutz (BMJV). Gesetz zur Verhütung und Bekämpfung von Infektionskrankheiten beim Menschen (Infektionsschutzgesetz - IfSG). [Act for the prevention and control of human infectious diseases (Protection against infection act)]. Berlin: BMJV; 2020. German. Available from: https://www.gesetze-im-internet.de/ifsg/

[12] Shen Y Zheng F Sun D Ling Y Chen J Li F Epidemiology and clinical course of COVID-19 in Shanghai, China. Emerg Microbes Infect. 2020;9(1):1537-45. 10.1080/22221751.2020.1787103 32573353 PMC7473125

[13] Guan WJ Ni ZY Hu Y Liang WH Ou CQ He JX Clinical Characteristics of Coronavirus Disease 2019 in China. N Engl J Med. 2020;382(18):1708-20. 10.1056/NEJMoa2002032 32109013 PMC7092819

[14] Wang D Yin Y Hu C Liu X Zhang X Zhou S Clinical course and outcome of 107 patients infected with the novel coronavirus, SARS-CoV-2, discharged from two hospitals in Wuhan, China. Crit Care. 2020;24(1):188. 10.1186/s13054-020-02895-6 32354360 PMC7192564

[15] Tostmann A Bradley J Bousema T Yiek WK Holwerda M Bleeker-Rovers C Strong associations and moderate predictive value of early symptoms for SARS-CoV-2 test positivity among healthcare workers, the Netherlands, March 2020. Euro Surveill. 2020;25(16):2000508. 10.2807/1560-7917.ES.2020.25.16.2000508 32347200 PMC7189649

[16] Sudre CH Lee KA Lochlainn MN Varsavsky T Murray B Graham MS Symptom clusters in COVID-19: A potential clinical prediction tool from the COVID Symptom Study app. Sci Adv. 2021;7(12):eabd4177. 10.1126/sciadv.abd4177 33741586 PMC7978420

[17] Struyf T Deeks JJ Dinnes J Takwoingi Y Davenport C Leeflang MM Signs and symptoms to determine if a patient presenting in primary care or hospital outpatient settings has COVID-19 disease. Cochrane Database Syst Rev. 2020;7:CD013665. 32633856 10.1002/14651858.CD013665 PMC7386785

[18] European Centre for Disease Prevention and Control (ECDC). Case definition for coronavirus disease 2019 (COVID-19). Stockholm: ECDC. [Accessed: 1 Mar 2020]. Available from: https://www.ecdc.europa.eu/en/covid-19/surveillance/case-definition

[19] European Centre for Disease Prevention and Control (ECDC). Guidance for discharge and ending isolation in the context of widespread community transmission of COVID-19. Stockholm: ECDC. 8 April 2020. Available from: https://www.ecdc.europa.eu/sites/default/files/documents/covid-19-guidance-discharge-and-ending-isolation-first%20update.pdf

[20] Ferrucci R Dini M Groppo E Rosci C Reitano MR Bai F Long-Lasting Cognitive Abnormalities after COVID-19. Brain Sci. 2021;11(2):235. 10.3390/brainsci11020235 33668456 PMC7917789

[21] Hugon J Msika EF Queneau M Farid K Paquet C. Long COVID: cognitive complaints (brain fog) and dysfunction of the cingulate cortex. J Neurol. 2021;1-3. 34143277 10.1007/s00415-021-10655-x PMC8211714

[22] Carfì A Bernabei R Landi F Gemelli Against COVID-19 Post-Acute Care Study Group. Persistent Symptoms in Patients After Acute COVID-19. JAMA. 2020;324(6):603-5. 10.1001/jama.2020.12603 32644129 PMC7349096

[23] Tenforde MW Kim SS Lindsell CJ Billig Rose E Shapiro NI Files DC Symptom Duration and Risk Factors for Delayed Return to Usual Health Among Outpatients with COVID-19 in a Multistate Health Care Systems Network - United States, March-June 2020. MMWR Morb Mortal Wkly Rep. 2020;69(30):993-8. 10.15585/mmwr.mm6930e1 32730238 PMC7392393

[24] Halpin SJ McIvor C Whyatt G Adams A Harvey O McLean L Post-discharge symptoms and rehabilitation needs in survivors of COVID-19 infection: a cross-sectional evaluation. J Med Virol. 2020. 10.1002/jmv.26368 32729939

[25] Mahase E. Covid-19: What do we know about "long covid"? BMJ. 2020;370:m2815. 10.1136/bmj.m2815 32665317

[26] Federal Ministry of Health. Verordnung zum Anspruch auf bestimmte Testungen für den Nachweis des Vorliegens einer Infektion mit dem Coronavirus SARS-CoV-2. [Ordinance on the right to certain tests to prove the presence of an infection with the SARS-CoV-2 coronavirus]. Berlin: Federal Ministry of Health. 8 Jun 2020. German. Available from: https://www.bundesanzeiger.de/pub/publication/tStb7Z9SHEGX5Lep0Ha/content/tStb7Z9SHEGX5Lep0Ha/BAnz%20AT%2009.06.2020%20V1.pdf?inline

