# Supplementary material for: A longitudinal study on symptom duration and 60-day clinical course in non-hospitalised COVID-19 cases in Berlin, Germany, March to May, 2020
Source: Euro Surveill. 2021 Oct 28;26(43):2001757. doi: 10.2807/1560-7917.ES.2021.26.43.2001757 (PMC8555372; doi:10.2807/1560-7917.ES.2021.26.43.2001757)
Supplement: Supplementary Material [file 20-01757_MULLER_Supplement.pdf]

Supplementary materials

Disclaimer

This supplementary material is hosted by *Eurosurveillance* as supporting information alongside the article 'A longitudinal study on symptom duration and 60-day clinical course in non-hospitalised COVID-19 cases in Berlin, Germany, March to May, 2020', on behalf of the authors, who remain responsible for the accuracy and appropriateness of the content. The same standards for ethics, copyright, attributions and permissions as for the article apply. Supplements are not edited by *Eurosurveillance* and the journal is not responsible for the maintenance of any links or email addresses provided therein.

Label	Description	Page number
S1:	Additional information on the study setting	2
S2:	Flowchart depicting inclusion criteria and number of cases interviewed	3
S3:	Investigation form (day 1-14, day 30)	4
S4:	Applied definitions of symptoms	5
S5:	Difference between sexes in symptoms	7
S6:	Symptoms among COVID-19 patients at day 30 and day 60 after symptom onset who had experienced these symptoms already before	8

S1: Additional information on the study setting

Study area

With 3.7 million inhabitants, Berlin is the most densely populated state in Germany. Physicians and laboratories notify clinically suspect and laboratory-confirmed cases of COVID-19 directly to the local health authority (LHA) of residence of the case. With a population of 370,000, Mitte is the district with the second largest population in the state [1].

Public health activities on COVID-19 in the study area

First cases of COVID-19 were reported to the LHA in Mitte district in the beginning of March 2020. In accordance with the recommendations by the German national public health institute (Robert Koch Institute, RKI), a case was defined as a person with detection of SARS-CoV-2 by PCR in nasopharyngeal or oropharyngeal swab or any other body fluid. Initial exploratory interviews with cases were conducted by the LHA by telephone to assess details on contact persons, potential exposures, risk factors of infection, onset and prevalence of symptoms. At the time of sampling, home isolation of cases was required for a period of at least 14 days. Cases could end the isolation period if they were symptom-free for a minimum of two consecutive days at the end of the initial 14 days.

Public health testing recommendations during the study period

The testing strategy as recommended by Robert Koch Institute (RKI) changed during the study period. From the beginning of the study up to early May, testing for symptomatic patients lacking a known contact to a laboratory confirmed case was only recommended as part of a differential diagnostic work-up, especially for medical staff or patients with known risk factors for a fulminant disease course. Since early May, 2020, testing was expanded to all symptomatic patients including patients presenting with dysosmia or dysgeusia, regardless of their epidemiological link, their occupational activity or their pre-existing condition. Testing was always free of charge (2).

References

1. *Statistischer Bericht A I 5 – hj 1 / 17, Einwohnerinnen und Einwohner im Land Berlin am 30. Juni 2017. 2017, Amt für Statistik Berlin-Brandenburg: Potsdam.*

2. *Robert Koch Institute: COVID-19 Verdacht: Testkriterien und Maßnahmen (Testing criteria and measures in pressumed COVID-19 cases). Available on: https://www.rki.de/DE/Content/InfAZ/N/Neuartiges_Coronavirus/Massnahmen_Verdachtsfall_Infografik_DINA3.pdf?__blob=publicationFile*

S2: Flowchart depicting inclusion criteria and number of cases interviewed, Berlin, Germany, March to May 2020

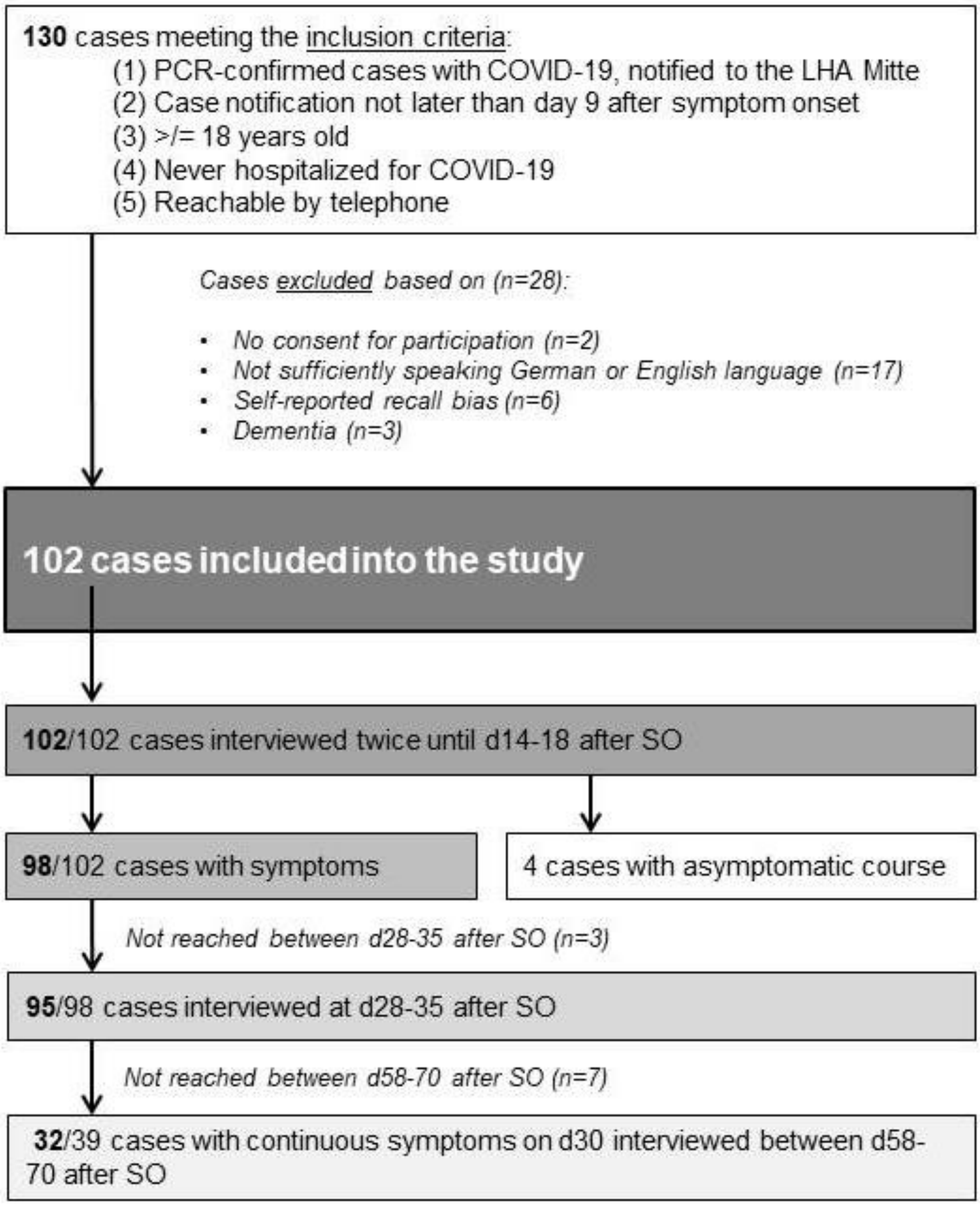

Legend:
Sampling period from March 30, 2020 to April 9, 2020 and April 13, 2020 to May 20, 2020
SO = symptom onset

S3: Investigation form (day 1-18, day 30) to assess symptom duration in COVID-19 patients, Berlin, Germany, March to May 2020. The investigation form was in German as telephone interviews were conducted in German. To facilitate interpretation for non-German readers we have labelled the form. Section (A) asks about personal information of the patient (name, date of birth, telephone number). Section (B) refers to demographic, smoking and anthropometric information. Section (C) is for the initials of the interviewer by interview data as well as the exact date of the interview. Section (D) records any comorbidities and use of medication. Section (E) collects information on presence/absence of symptoms (rows) by time (columns) as well as symptom severity for some symptoms. Section (F) ask about hospitalisation, duration and name of the hospital.

Befragung: Klinischer Verlauf bei COVID-19

C
Interviewer: **d7-9:** **d14-16:** **d28-30:**
Datum: **d7-9:** **d14-16:** **d28-30:**

A
ID:
Name:
Vorname:
Geburtsdatum:
Telefonnummer:

B
Geschlecht (m/w):
Aktiver Raucher (Zig/Woche/py):
Ex-Raucher (py):
Gewicht:
Größe:

D
Vorerkrankungen: KEINE ○ COPD ○ KHK ○ NI ○ Nervenerkr. ○
Asthma ○ Herzinsuff ○ Sonstige:
Medikation:
Immuntherapie (aktuell, Vergangenheit)?

E

Tag des Interviews	○	○	○	○	○	○	○	○	○	○	○	○	○	○	○	○	○	○	○
**NA (7) Weiss nicht (8) Missing (9)**	1.Tag	2.Tag	3. Tag	4. Tag	5. Tag	6. Tag	7. Tag	8. Tag	9. Tag	10. Tag	11. Tag	12. Tag	13. Tag	14. Tag	15. Tag	16. Tag	17. Tag	18. Tag	28.-30. Tag
**Wochentag**																			
**Datum:**	/	/	/	/	/	/	/	/	/	/	/	/	/	/	/	/	/	/	/
1. **Fieber:** nein (0), ja, aber nicht gemessen (1), höchste gemessene Temperatur: °Celsius-Angabe																			
1.1. **Einnahme Fiebersenker (welche:QS)?** nein (0) ja (1)																			
2. **Schüttelfrost:** nein (0) ja (1)																			
3. **Husten:** nein (0) trocken (1) feucht (2) blutig (3)																			
4. **Halssymptome:** nein (0) Kratzen (1) Schmerzen (2)																			
5. **Heiserkeit:** nein (0) ja (1)																			
6. **Schnupfen:** nein (0) ja, verstopft (1), ja, triefend (2)																			
7. **Luftnot:** nein (0) In Ruhe (1) Belastung (2)																			
8. Kopfschmerzen: nein (0) ja (1)																			
8.1. **Kopfschmerzen (Qualität):** dumpf (1) pochend (2) stechend (3) undiff 4)																			
8.2. **Kopfschmerzen (Lok.):** frontal (1) nuchal (2) holocephal (3) undiff (4)																			
8.3. **Kopf-Sx VAS**																			
9. **Rückenschmerzen:** nein (0) BWS (1) LWS (2) gesamt (3)																			
10. **Muskel-/Gliederschmerzen:** nein (0) ja (1)																			
11. **Brustschmerzen:** nein (0) AP (1) Pleuritisch (2) undiff (3)																			
12. **Gelenkschmerzen: (ggf. QS)** nein (0) ja (1)																			
13. **Bauchschmerzen:** nein (0) Krampfartig (1) Dumpf (2) undiff (3)																			
13.1. **Bauchschmerzen Lok:** Oberbauch (1) Unterbauch (2) Gesamt (3)																			
13.2. **Bauchschmerzen VAS**																			
14 **Stuhlauffälligkeiten:** nein (0) wässrig(1) breiig (2) blutig (3)																			
14.1. **Stuhlauffälligkeiten:** Wie oft?																			
15. **Übelkeit:** nein (0) ja (1)																			
16. **Erbrechen:** nein (0) wie oft:																			
17. **Fatigue (Müdigkeit):** nein (0) ja (1)																			
18. **Antriebslosigkeit:** nein (0) ja (1)																			
19. **Geruchsminderung: (Beispielgerüche: Kaffee, Desinfektionsmittel)** nein (0) mild (1) mäßig (2) total (3)																			
20. **Geschmacksminderung (salzig, bitter, sauer, süß):** nein (0) mild (1) mäßig (2) total (3)																			
21. **Taubheitsgefühl Hände & Füße** nein (0) ja (1)																			
22. **Hautausschlag (ggf. QS):** nein (0) juckend (1) nicht juckend (2)																			
23. **Konjunktivitis:** nein (0) ja (1)																			
24. **Krankheitsgefühl** nein (0) mild (1) mäßig (2) stark (3)																			
25. **Hyperästhesie? (QS)** nein (0) ja (1)																			
26. **Sonstige Symptome (welche):** nein (0) ja (1)																			

F
Wurden Sie wegen COVID im Krankenhaus behandelt (erst "Nein" nach letztem Interview ankreuzen)? nein (0) ○ ja (1) ○ **wenn ja:** Beginn: Ende:
Name des Krankenhauses: Station:

S4: Applied definitions of symptoms in COVID-19 patients, Berlin, Germany, March to May 2020

**Symptom:**	**Definition:**
**Fever**	Self-reported OR measured body temperature =/>38,0°C at least once
**Antpyretic agent**	Acetylsalicylic acid, ibuprofene, diclofenac, acetaminophen, metamizole, etc.
**Chills and rigor**	Chills and rigor affecting the whole body
**Cough**	In chronic cough: increase in the frequency or change in the quality of the cough
**Sore throat**	Sore throat OR pain while speaking or swallowing
**Hoarseness**	Voice strongly weakened
**Rhinitis**	Catarrh („stuffy nose“) with OR without secretion, if both are present the more prominent symptom
**Dyspnea**	Shortness of breath (independent of stuffy nose)
**Headache**	
**Back pain**	
**Chest pain**	Pleuritic pain (breath-related pain) OR tightness of the chest on exertion OR feeling of pressure on the chest
**Joint pain**	
**Abdominal pain**	
**Changes in bowel habit, diarrhoea**	Loose stools independent of frequency
**Nausea**	
**Vomiting**	
**Fatigue**	Extreme tiredness or need for sleep without any sleep deficit and no improvement after sleep
**Listlessness**	Loss of drive
**Dysosmia**	Impairment of the sense of smell (independent of stuffy nose)
**Dysgeusia**	Impairment of at least one of the taste modalities: sweetness, sourness, saltiness, bitterness, savoriness (umami)
**Muscle pain**	
**Numbness of upper and/or lower limbs**	Decrease or loss of sensation in upper AND/OR lower limbs
**Skin rash**	
**Conjunctivitis**	Prolonged redness of the eyes
**Hyperaesthesia**	Severe hypersensitivity of the skin (e.g. sunburn-like feeling)
**Reduced physical performance capacity**	Decrease in physical AND/OR psychological performance in comparison to before COVID-19

S5: Difference between sexes in COVID-19 symptoms, Berlin, Germany, March to May 2020

	Day 1-14			Day 30			Day 60		
**Symptoms**	**Female** N = 57	**Male** N = 41	**P-value**	**Female** N = 55	**Male** N = 40	**P-value**	**Female** N = 53	**Male** N = 35	**P-value**
**Malaise**	54 (95%)	38 (93%)	0.7	6 (11%)	1 (2.5%)	0.2	3 (5.7%)	0 (0%)	0.4
**Headache**	44 (77%)	26 (63%)	0.2	5 (9.1%)	1 (2.5%)	0.4	2 (3.8%)	0 (0%)	0.7
**Rhinitis**	39 (68%)	29 (71%)	>0.9	2 (3.6%)	1 (2.5%)	>0.9	2 (3.8%)	0 (0%)	0.7
**Muscle pain**	39 (68%)	25 (61%)	0.6	0 (0%)	0 (0%)	-	1 (1.9%)	0 (0%)	>0.9
**Cough**	36 (63%)	27 (66%)	>0.9	7 (13%)	4 (10%)	0.8	2 (3.8%)	1 (2.9%)	>0.9
**Dysosmia**	34 (60%)	25 (61%)	>0.9	14 (25%)	7 (18%)	0.5	9 (17%)	3 (8.6%)	0.4
**Fatigue**	32 (56%)	21 (51%)	0.8	3 (5.5%)	2 (5.0%)	>0.9	3 (5.7%)	0 (0%)	0.4
**Sore throat**	28 (49%)	19 (46%)	>0.9	3 (5.5%)	0 (0%)	0.3	1 (1.9%)	0 (0%)	>0.9
**Dysgeusia**	31 (54%)	14 (34%)	0.075	5 (9.1%)	3 (7.5%)	>0.9	3 (5.7%)	2 (5.7%)	>0.9
**Fever**	22 (39%)	16 (39%)	>0.9	0 (0%)	0 (0%)	-	0 (0%)	0 (0%)	-
**Back pain**	22 (39%)	14 (34%)	0.8	3 (5.5%)	0 (0%)	0.3	1 (1.9%)	0 (0%)	>0.9
**Chills**	16 (28%)	15 (37%)	0.5	3 (5.5%)	0 (0%)	0.3	2 (3.8%)	1 (2.9%)	>0.9
**Dyspnea**	17 (30%)	11 (27%)	>0.9	0 (0%)	0 (0%)	-	0 (0%)	0 (0%)	-
**Nausea**	22 (39%)	5 (12%)	0.008	1 (1.8%)	0 (0%)	>0.9	1 (1.9%)	0 (0%)	>0.9
**Diarrhoea**	13 (23%)	14 (34%)	0.3	2 (3.6%)	0 (0%)	0.5	0 (0%)	0 (0%)	-
**Joint pain**	10 (18%)	10 (24%)	0.6	0 (0%)	0 (0%)	-	1 (1.9%)	0 (0%)	>0.9
**Abdominal pain**	11 (19%)	6 (15%)	0.7	1 (1.8%)	0 (0%)	>0.9	0 (0%)	0 (0%)	-
**Chest tightness**	12 (21%)	4 (9.8%)	0.2	3 (5.5%)	0 (0%)	0.3	1 (1.9%)	0 (0%)	>0.9
**Listlessness**	12 (21%)	4 (9.8%)	0.2	2 (3.6%)	0 (0%)	0.5	0 (0%)	0 (0%)	-
**Hyperesthesia**	10 (18%)	7 (17%)	>0.9	0 (0%)	0 (0%)	-	0 (0%)	0 (0%)	-
**Pleuritic pain**	7 (12%)	2 (4.9%)	0.3	0 (0%)	0 (0%)	-	0 (0%)	0 (0%)	-
**Hoarseness**	5 (8.8%)	3 (7.3%)	>0.9	0 (0%)	0 (0%)	-	0 (0%)	0 (0%)	-
**Rash**	4 (7.0%)	2 (4.9%)	>0.9	0 (0%)	0 (0%)	-	0 (0%)	0 (0%)	-
**Vomiting**	4 (7.0%)	2 (4.9%)	>0.9	0 (0%)	0 (0%)	-	0 (0%)	0 (0%)	-
**Chest pain, unspecific**	3 (5.3%)	2 (4.9%)	>0.9	1 (1.8%)	0 (0%)	>0.9	0 (0%)	0 (0%)	-
**Numbness**	3 (5.3%)	2 (4.9%)	>0.9	0 (0%)	0 (0%)	-	0 (0%)	0 (0%)	-
**Conjunctivitis**	1 (1.8%)	2 (4.9%)	0.6	0 (0%)	0 (0%)	-	0 (0%)	0 (0%)	-

S6: Proportion of COVID-19 patients at day 30 and day 60 after symptom onset who had symptoms already experienced prior, Berlin, Germany, March to May 2020

Symptoms:	Day 30<br>N = 39	Day 60<br>N = 18
**Abdominal pain**	1 (100%)	0 (0%)
**Back pain**	3 (100%)	1 (100%)
**Chest tightness**	3 (100%)	1 (100%)
**Chest pain, unspecific**	0 (0%)	0 (0%)
**Rhinitis**	3 (100%)	2 (100%)
**Cough**	10 (91%)	3 (100%)
**Diarrhoea**	2 (100%)	0 (0%)
**Dysgeusia**	8 (100%)	4 (80%)
**Dysosmia**	20 (95%)	12 (100%)
**Dyspnea**	1 (33%)	2 (67%)
**Fatigue**	5 (100%)	3 (100%)
**Listlessness**	1 (50%)	0 (0%)
**Headache**	6 (100%)	2 (100%)
**Joint pain**	0 (0%)	0 (0%)
**Malaise**	7 (100%)	3 (100%)
**Muscle pain**	0 (0%)	0 (0%)
**Nausea**	1 (100%)	1 (100%)
**Sore throat**	3 (100%)	0 (0%)
